# Delineation of Mitochondrial DNA Variants From Exome Sequencing Data and Association of Haplogroups With Obesity in Kuwait

**DOI:** 10.3389/fgene.2021.626260

**Published:** 2021-02-11

**Authors:** Mohammed Dashti, Hussain Alsaleh, Muthukrishnan Eaaswarkhanth, Sumi Elsa John, Rasheeba Nizam, Motasem Melhem, Prashantha Hebbar, Prem Sharma, Fahd Al-Mulla, Thangavel Alphonse Thanaraj

**Affiliations:** ^1^Genetics and Bioinformatics Department, Dasman Diabetes Institute, Kuwait City, Kuwait; ^2^Kuwait Identification DNA Laboratory, General Department of Criminal Evidence, Ministry of Interior, Kuwait City, Kuwait; ^3^Department Special Services Facilities, Dasman Diabetes Institute, Kuwait City, Kuwait

**Keywords:** mitochondrial, DNA, haplogroup, exome, obesity

## Abstract

**Background/Objectives:**

Whole-exome sequencing is a valuable tool to determine genetic variations that are associated with rare and common health conditions. A limited number of studies demonstrated that mitochondrial DNA can be captured using whole-exome sequencing. Previous studies have suggested that mitochondrial DNA variants and haplogroup lineages are associated with obesity. Therefore, we investigated the role of mitochondrial variants and haplogroups contributing to the risk of obesity in Arabs in Kuwait using exome sequencing data.

**Subjects/Methods:**

Indirect mitochondrial genomes were extracted from exome sequencing data from 288 unrelated native Arab individuals from Kuwait. The cohort was divided into obese [body mass index (BMI) ≥ 30 kg/m^2^] and non-obese (BMI < 30 kg/m^2^) groups. Mitochondrial variants were identified, and haplogroups were classified and compared with other sequencing technologies. Statistical analysis was performed to determine associations and identify mitochondrial variants and haplogroups affecting obesity.

**Results:**

Haplogroup R showed a protective effect on obesity [odds ratio (OR) = 0.311; *P* = 0.006], whereas haplogroup L individuals were at high risk of obesity (OR = 2.285; *P* = 0.046). Significant differences in mitochondrial variants between the obese and non-obese groups were mainly haplogroup-defining mutations and were involved in processes in energy generation. The majority of mitochondrial variants and haplogroups extracted from exome were in agreement with technical replica from Sanger and whole-genome sequencing.

**Conclusions:**

This is the first to utilize whole-exome data to extract entire mitochondrial haplogroups to study its association with obesity in an Arab population.

## Introduction

Mitochondria play a role in generating cellular energy via oxidative phosphorylation (OXPHOS), reactive oxygen species production, and apoptosis. Human mitochondrial DNA (mtDNA) is circular, double-stranded, and 16,569 base pairs (bp) in size and contains 37 genes that code for 22 transfer RNAs, two ribosomal RNAs that are necessary for protein synthesis, and 13 messenger RNAs that are required for OXPHOS (Anderson et al., 1981; Andrews et al., 1999). Each mitochondrion contains several copies of mtDNA, and each cell contains many mitochondria (Hosgood et al., 2010). mtDNA contains a major non-coding region called the control/D-loop region, which regulates mitochondrial transcription and replication. The mitochondrial control region is located at mitochondrial nucleotide positions 16,024–576 and is susceptible to a high rate of mtDNA alterations, particularly at the hypervariable regions (Greenberg et al., 1983) as well as under conditions of increased oxidative stress (Clayton, 2000). mtDNA variants are maternally inherited without recombination and may accumulate over time. A mitochondrial haplogroup is a group of individuals who share the same accumulated mtDNA variants and can be geographically restricted, making then traceable via maternal linage. Different haplogroups form diverse branches of a mitochondrial phylogenetic tree. The sub-Saharan Africans are characterized by L0–L6; the South Asians by haplogroups R5–R8, M2–M6, and M4–67; the Europeans, Southwest Asians, and North Africans by haplogroups U, HV, JT, N1, N2, and X; and the East Asians by haplogroups A–G, Z, and M7–M9 (Loogvali et al., 2004; Chaubey et al., 2007; Soares et al., 2010; Kivisild, 2015).

Sanger sequencing is considered the gold standard for detecting mtDNA variants. This approach has progressed to next-generation sequencing (NGS) platform, as it provides high-throughput sequence data for large cohort studies and is less labor-intensive and time-consuming than Sanger sequencing (Calvo et al., 2012; Chinnery et al., 2012; Tang et al., 2013; Wong, 2013). Recently, a number of studies demonstrated that whole-genome sequencing and off-target exome sequencing are able to target both nuclear DNA and mtDNA for the diagnosis of monogenic cases and association studies for multifactorial disorders (Picardi and Pesole, 2012; Delmiro et al., 2013; Samuels et al., 2013; Griffin et al., 2014; Li et al., 2014). Particularly interesting studies include the following: Wagner et al. (2019) evaluated if mtDNA analysis can be performed using exome data; Diroma et al. (2014) extracted mtDNA sequences from exome data to reconstruct human population history using mtDNA variant as marker and to illustrate the involvement of mtDNA in pathology; and Patowary et al. (2017) analyzed the mtDNA sequence derived from whole-exome sequencing and studied haplogroup and variant association in autism spectrum disorder. Nevertheless, to the best of our knowledge, there is no demonstration in the literature on the efficiency of mitochondrial variant calling from whole genome and exome data when compared with the calling using Sanger sequencing data. In addition, there are no studies on mitochondrial haplogroup and variant association with obesity using exome data with potential significant results.

Obesity has become a worldwide epidemic, particularly among Arab populations. In Kuwait, the prevalence of obesity ranges from 37 to 50% (Ng et al., 2014; World Health Organization [WHO], 2018). While obesity has a large heritable component, elucidating these determinants is complicated by the complex interplay between environmental, behavioral, and genetic factors. Genetic studies into obesity have identified monogenic genes using linkage analysis and common variants using genome-wide association studies (GWASs) (Ramachandrappa and Farooqi, 2011). However, obesity-associated genetic loci are often identified in nuclear DNA and have a modest effect that cannot explain the high heritability estimates, and well-defined genetic loci are often from rare familial syndromes (Stunkard et al., 1990; Bouchard and Perusse, 1993; Sorensen et al., 1998).

Mitochondrial variants, haplogroups, and copy number variations have been proposed as potential causative or protective factors for complex and multifactorial disorders and can explain the missing heritability for obesity.

Several studies have shown correlations between mitochondrial function and obesity (Wortmann et al., 2009; Fernandez-Sanchez et al., 2011; Naukkarinen et al., 2014). These findings have led to studies that have evaluated whether mitochondrial dysfunction in obesity is due to inherited sequence variations. Several mitochondrial variants and haplogroups have been associated with obesity in different ethnicities (Yang et al., 2011; Grant et al., 2012; Nardelli et al., 2013; Flaquer et al., 2014; Knoll et al., 2014; Ebner et al., 2015; Veronese et al., 2018; Eaaswarkhanth et al., 2019). The genotyping data used in these studies were from the mitochondrial control region (Nardelli et al., 2013; Ebner et al., 2015; Veronese et al., 2018; Eaaswarkhanth et al., 2019) and/or extracted from GWASs (Yang et al., 2011; Grant et al., 2012; Flaquer et al., 2014; Knoll et al., 2014).

The present study investigates the role of mitochondrial variants and haplogroups contributing to the risk of obesity in Arabs in Kuwait. Arabian Peninsula populations present unique features in the context of the worldwide genetic diversity (Alsmadi et al., 2013, 2014; Thareja et al., 2015): (1) they resulted from an old and continuous admixture between African, European, and Asian ancestries; (2) the high level of consanguineous mating increases frequencies of rare variants and extends stretches of homozygous chromosomal fragments. Further, the Arabian Peninsula, by virtue of being the exit point for the Southern Route of Africa, was indeed the first staging post in the spread of modern humans around the world (Fernandes et al., 2012). Hence, the characterization of Arabian exome variant data potentiates the easy detection of functional variants, contributing information to discover new disease mechanisms.

The present study examined the association of mitochondrial haplogroups and variants with obesity using off-target whole-exome data from a Kuwaiti population. The study used whole-genome data and Sanger sequencing data as quality control samples for mitochondrial variants called from exome reads.

## Materials and Methods

### Exome Data

We analyzed 288 exomes from Kuwaiti individuals who were included in a previously published study (John et al., 2018). Samples from the individuals were divided into two groups according to body mass index (BMI): obese (BMI ≥ 30 kg/m^2^; *n* = 152) and non-obese (<30 kg/m^2^; n = 136). Samples were sequenced using two different exome kits: samples from 160 individuals were sequenced using the TruSeq Exome Enrichment kit, and samples from the remaining 128 individuals were sequenced using the Nextera Rapid Capture Exome kit, both using the Illumina HiSeq platform (Illumina Inc. United States) (John et al., 2018). Target files of both exome kits show that both contain the same 11 mtDNA regions where each target region covers an average of 1,000 bp. Whole genomes from three of the 288 individuals were sequenced in our previous studies (Alsmadi et al., 2014; Thareja et al., 2015), and mtDNA sequences extracted from these individuals were used as quality control samples for mitochondrial variants called from exome reads. Furthermore, we previously sequenced mtDNA D-loops from 173 individuals using conventional DNA Sanger sequencing (Eaaswarkhanth et al., 2019), and variants called using the control regions were used as quality controls for mitochondrial variant calling using exome sequences.

### Mitochondrial DNA Sequences, Variant Calling, and Annotation

Raw paired-end reads (100 bp) from exome sequencing were mapped to human genome assembly GRCh37 using Burrows–Wheeler Aligner (BWA-MEM version v07-17) with default mapping options (Li, 2013). Duplicate reads were removed using Picard version 2.20.2^1^. The revised Cambridge Reference Sequence (rCRS) (Andrews et al., 1999) for human mtDNA as deposited in the GenBank NCBI database under accession number NC_012920.1 was extracted using SAMtools version 0.1.19 (Li et al., 2009), and the average mtDNA coverage was calculated using Genome Analysis Toolkit (GATK) version v3.8-1-0 (McKenna et al., 2010). mtDNA BAM files were generated for each sample. We subsequently used the GATK haplocaller with default parameters on the extracted mtDNA BAM files to generate variants for each sample in Genomic Variant Call Format (GVCF). All the GVCF files were combined into a single GVCF that was subsequently used to genotype the mtDNA variants. Annotation of the variants was performed using Ensembl Variant Effect Predictor (McLaren et al., 2016) and mitomap/mitomaster^2^ (Lott et al., 2013).

### Haplogroup Assignment

We used raw variant calling format files for whole mtDNA from 288 samples from Kuwaiti individuals and three whole-genome (technical replica) samples to determine their maternal haplogroups. Haplogroup calling was performed using HaploGrep 2.0 (Weissensteiner et al., 2016) based on PhyloTree Build 17 (accessed on 19 December 2019). To determine the accuracy of mitochondrial haplogroup prediction from exome data, we compared the results with a matched mitochondrial haplogroup profiling of 173 samples from Kuwaiti individuals whose mitochondrial control region variants were called using Sanger sequencing in our previously study (Eaaswarkhanth et al., 2019). We also assessed the agreement in assignment of major mitochondrial haplogroups between whole-exome and whole-genome samples. Further, the graphical phylogenetic trees for the haplogroups R and L were generated from HaploGrep 2.0. The Median-Joining networks of R and L haplogroups were constructed using PopART version 1.7 (Leigh and Bryant, 2015).

### Statistical Analyses

Statistical analysis of clinical characteristics was performed using R Project for Statistical Computing software (version 3.6.2)^3^. Quantitative clinical variables (assuming continuous values) were ascertained for normality assumption using Shapiro–Wilk test and presented as either mean ± standard deviation or median and interquartile range. Non-parametric Mann–Whitney *U* test was used to compare age and BMI scores (which may have skewed distribution) between obese and non-obese groups. In the cases of categorical variables, descriptive statistics were presented as number and percentage, and chi-square test was applied to find associations or significant differences between them.

The differences in the counts of non-synonymous over synonymous mutations between the R (protective) and L (risk) haplogroups were examined using Fisher exact test. Further, the significance of the differences in dN/dS ratio between the two haplogroups was calculated using unpaired Wilcoxon rank sum test available in R software.

Principal component analysis (PCA) was conducted to determine whether the mtDNA profiles could cluster the samples based on obese/non-obese categorizations and assigned haplogroups. We used the PCA tools package of the R software to perform the PCA. Fisher exact test was used to investigate the differences in the distribution of mitochondrial haplogroups and variants between the obese and non-obese groups. Additionally, logistic regression analysis was performed to determine haplogroup association (adjusted for age and sex) with traits using IBM SPSS statistical software (version 25). PLINK software (version 1.9) (Chang et al., 2015) was used to test mtDNA variant association (adjusted for age, sex, and maternal haplogroups) with traits. A two-tailed *P*-value < 0.05 was considered statistically significant.

## Results

### Study Population

Table 1 shows the descriptive statistics for the clinical characteristics of the study cohort and subcohorts of 152 (52.8%) obese and 136 (47.2%) non-obese Kuwaiti individuals. There were no significant differences between the obese and non-obese groups in terms of sex; however, the mean age was significantly higher in the obese group compared with the non-obese group (*P* > 0.001). This difference was in agreement with the results reported in our recent study on Arab population from Kuwait (Eaaswarkhanth et al., 2019). As expected, BMI was significantly different between obese and non-obese groups (*P* > 0.001).

**TABLE 1:** Clinical characteristics of the Kuwaiti study samples.

	**Obese**	**Non-obese**	**Total**	***P*-value for obese versus non-obese groups**
	**(*N* = 152) *n* (%)**	**(*N* = 136) *n* (%)**	**(*N* = 288) *n* (%)**	
**Gender**				
Male	54 (35.5%)	63 (46.3%)	117 (40.6%)	0.08
Female	98 (64.5%)	73 (53.7%)	171 (59.4%)	
**Age (years)**				
≤25	4 (63.4%)	7 (63.6%)	6 (2.1%)	2.90E-11
26–34	6 (10.5%)	51 (89.5%)	57 (19.8%)	
35–44	21 (45.6%)	25 (54.3%)	46 (15.9%)	
≥45	121 (69.5%)	53 (30.5%)	174 (60.4%)	
Mean ± SD	57.1 ± 14.4	43.8 ± 16.7	50.8 ± 16.8	
Median (IQ)	58.5 (48–66.25)	36.5 (31–57.5)	52.5 (35–64)	
**BMI score**				
Mean ± SD	39.5 ± 6.8	24.8 ± 2.9	32.5 ± 9.1	<2.2E-16
Median (IQ)	38.5 (33.8–43.7)	24.8 (22.6–27.1)	30.9 (25.2–38.8)	

**P*-value for age categories for obese versus non-obese groups were calculated using Mann–Whitney *U* test. *P*-value for sex counts in obese versus non-obese groups were calculated using Chi-sq test. Abbreviations: BMI, body mass index; *N*, number of individuals; SD, standard deviation; IQ, inter-quartile.*

### Mitochondrial DNA Coverage and Variants

The average coverage of extracted mtDNA sequences from off-target whole-exome samples in our study cohort was 50 × using Nextera Rapid Capture Exome kit and 8 × using TruSeq Exome Enrichment kit. The average coverage of mtDNA sequences from whole-genome (technical replica) samples was 2,491×. The coverage of mtDNA sequences was expected to be high using whole-genome sequencing as the large number of mitochondria present in the cytoplasm contributed to a greater number of reads.

A total of 1,241 mtDNA single-nucleotide polymorphisms (SNPs) and insertion/deletion (INDELs) variants were identified among the 288 whole-exome samples. A comparison of the detected mtDNA variants (SNPs only) from whole-exome data with variants called from the Sanger sequenced reads for the corresponding samples revealed that 77% of the variants were common. Nevertheless, a higher detection rate of variants was observed in Nextera Rapid Capture Exome kit samples (87%) compared with the TruSeq Exome Enrichment kit samples (70%).

Some variants were detected by only Sanger sequencing—such variants were MT:71, 209, 235, 311, 315, 398, 411, 523, 524, 571, 573, 582, 16086, 16186, 16188, 16207, 16217, 16249, 16256, 16293, 16351, and 16399. On the other hand, some mtDNA variants (such as MT:513 and MT:16183) were detected only in whole-exome data. Inconsistencies were seen in the called genotypes at MT:302, MT:309, and MT:310 between Sanger and exome sequencing. Furthermore, all the mtDNA variants identified from whole exomes were detected in whole genomes of the same samples. Only four variants (including a mtDNA variant at position MT:3492) were detected in the whole genomes, but not in whole exomes.

### Mitochondrial Haplogroups Associated With Obesity

**FIGURE 1:**
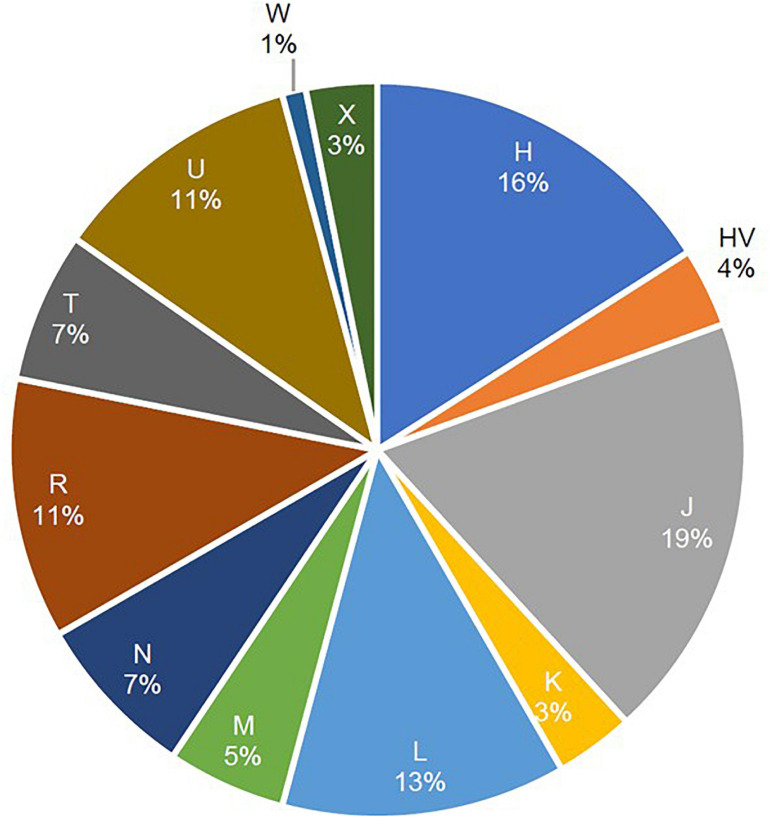
Frequencies of mitochondrial haplogroups in the study cohort of 288 Arab individuals from Kuwait.

A total of 12 maternal haplogroups (H, HV, J, K, L, M, N, R, T, U, W, and X) were identified from the mitochondrial variants extracted from the whole-exome samples. The most common maternal haplogroups among the 288 Kuwaiti individuals were J (19%), H (16%) L (13%), R (11%), and U (11%) (Figure 1). Good concordance was observed in haplogroup calling using whole exomes versus whole genomes versus Sanger sequenced reads. Among the 173 samples used for both exome sequencing and Sanger sequencing, 123 had the same major maternal haplogroups detected in both exomes and Sanger sequences (Supplementary Table 1). Further, same haplogroups (even at the resolution of subclade) were detected in both exomes and whole genomes in three samples that were analyzed using both sequencing techniques. One sample that had the same mitochondrial haplogroup detected using whole-exome and whole-genome sequencing differed in Sanger sequencing reads.

**FIGURE 2:**
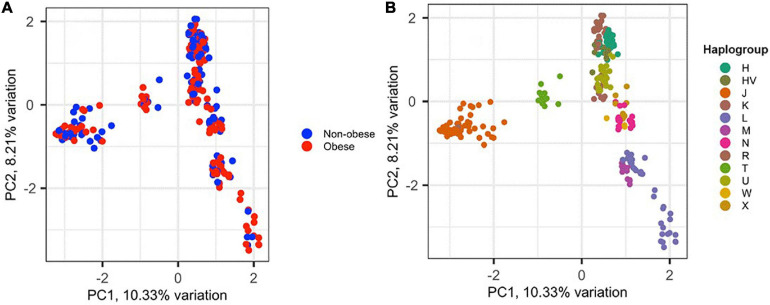
Principal component analysis (PCA) of the 288 Kuwaiti samples based on their mtDNA. **(A)** The two colors represent obese and non-obese samples, and the colors on **(B)** represent haplogroup origin of each sample. PC1 and PC2 on the *x*- and *y*-axes represent principal component 1 and principal component 2 and their variations in percentage, respectively.

To assess the amount of variation observed in mtDNA that could be attributed to BMI classification, PCA was performed for the 288 samples, in which 152 (52.8%) were classified as obese and 136 (47.2%) were non-obese (Figure 2). The samples did not cluster based on BMI classification or sex (data not shown), which may indicate that the genetic heritability of obesity in mtDNA is overestimated and/or our data are too small to demonstrate (Figure 2A). However, the samples clustered well based on haplogroup origin, emphasizing the importance of mtDNA when studying the maternal relatedness between individuals and populations (Figure 2B).

**FIGURE 3:**
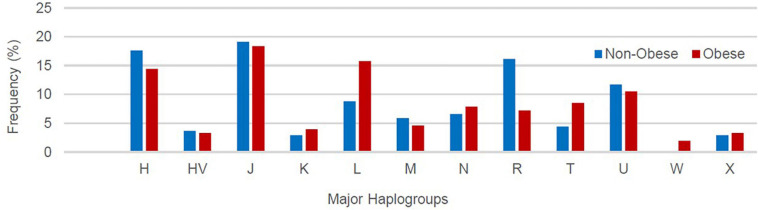
Frequency distribution of major mitochondrial DNA (mtDNA) haplogroups in obese and non-obese groups.

**FIGURE 4:**
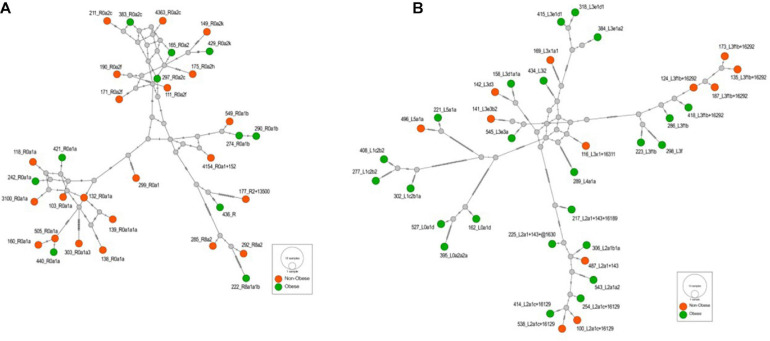
**(A)** The Median-Joining network of the haplogroup R that is associated with the reduced risk of obesity in the Kuwaiti population. The hatch marks on the edges denote nucleotide positions. **(B)** The Median-Joining network of the haplogroup L that is associated with the increased risk of obesity in the Kuwaiti population. The hatch marks on the edges denote nucleotide positions.

Supplementary Table 2 lists the variants used to assign haplogroup for each sample and the haplogroup assigned to the sample along with the HaploGrep 2 score (Weissensteiner et al., 2016). Table 2 shows the frequencies of each haplogroup in the obese and non-obese groups. The results indicated that individuals with the R haplogroup are at low risk of obesity [odds ratio (OR) = 0.4; *P* = 0.017)] and remained significant after adjusting the model for age and sex [OR = 0.311; 95% confidence interval (CI) = 0.135–0.717; and *P* = 0.006]. In addition, males with haplogroup R had a greater likelihood of being non-obese (OR = 4.84; *P* = 0.035) than obese (data not shown). On the other hand, haplogroup L individuals had a twofold increased risk of obesity (OR = 1.94), which was not significant (*P* = 0.074) but became significant after adjusting for age and sex using multivariate logistic regression (OR = 2.285; 95% CI = 1.02–5.14; and *P* = 0.046) (Table 2). The frequencies of haplogroups H and L differed significantly between obese and non-obese groups, where haplotype R was more frequent in the non-obese group and L was more frequent in the obese group (Figure 3). The complete phylogeny and Median-Joining networks of these obesity risk-associated haplogroups R and L along with their subclades in Kuwaiti individuals are presented in Supplementary Figures 1,2 and Figures 4A,B, respectively.

**TABLE 2:** Mitochondrial haplogroups associated with obesity in the Kuwaiti population.

**Haplogroup**	**Obese**	**Non-obese**	**OR**	***P*-value**	OR (95%CI)* after adjusting **the model for age and sex**	*P*-value* after adjusting the **model for age and sex**
	***N* (152)**	***N* (136)**				
H	22 (14.47%)	24 (17.65%)	0.79	0.463	0.786 (0.396–1.562)	0.492
HV	5 (3.29%)	5 (3.68%)	0.89	0.858	0.804 (0.211–3.064)	0.749
J	28 (18.42%)	26 (19.12%)	0.96	0.88	1.027 (0.539–1.958)	0.936
K	6 (3.95%)	4 (2.94%)	1.36	0.641	2.11 (0.504–8.829)	0.307
L	24 (15.79%)	12 (8.82%)	1.94	0.074	2.285 (1.015–5.141)	0.046
M	7 (4.61%)	8 (5.88%)	0.77	0.626	0.449 (0.143–1.415)	0.172
N	12 (7.89%)	9 (6.62%)	1.21	0.677	1.173 (0.434–3.173)	0.753
R	11 (7.24%)	22 (16.18%)	0.4	0.017	0.311 (0.135–0.717)	0.006
T	13 (8.55%)	6 (4.41%)	2.03	0.158	1.906 (0.657–5.532)	0.235
U	16 (10.53%)	16 (11.76%)	0.88	0.738	1.166 (0.509–2.672)	0.717
W	3 (1.97%)	0 (0%)	–	0.1	–	0.996
X	5 (3.29%)	4 (2.94%)	1.12	0.865	1.109 (0.255–4.835)	0.89

*Values after adjustment for age and gender. Abbreviations: N, number of individuals; OR, odds ratio; CI, confidence intervals for OR as calculated using logistic regression model using PLINK.

### dN/dS Ratio Between the R and L Haplogroups

**FIGURE 5:**
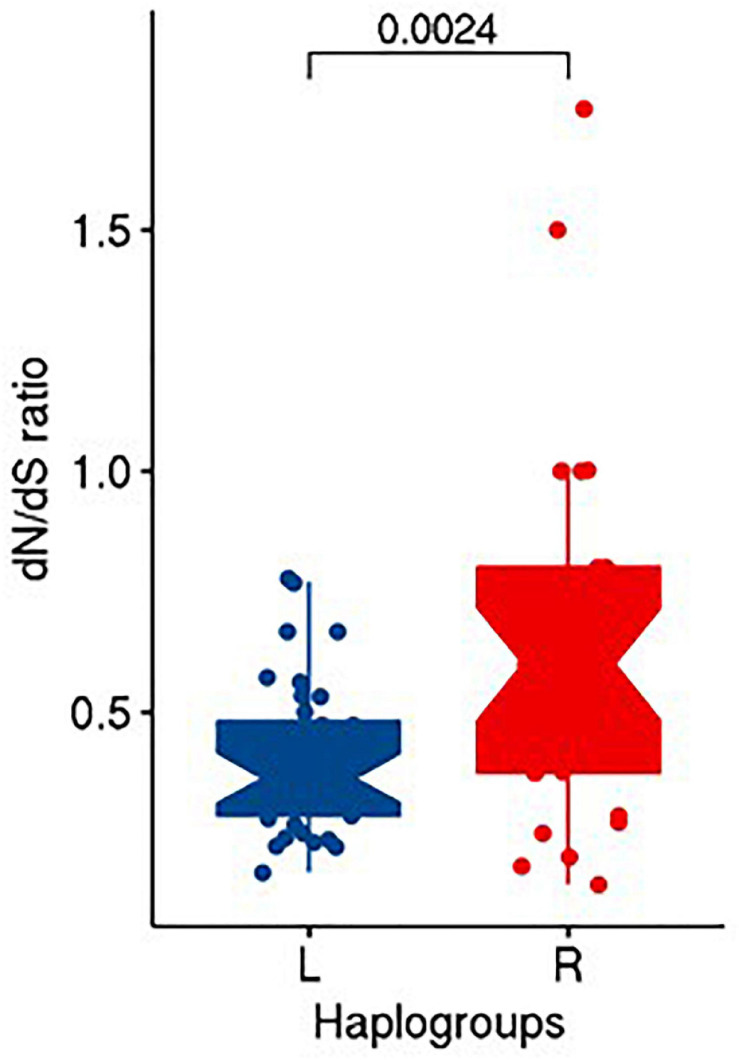
Distribution of dN/dS ratio in R and L haplogroups.

Upon performing Fisher exact test on the counts of non-synonymous and synonymous substitutions between R and L haplogroups, we did not find any significant difference (OR = 0.636; CI = 0.14–2.77; *P* = 0.547) in the distribution of non-synonymous and synonymous substitutions between them. However, upon computing the dN/dS ratios, we observed the median (IQR) of dN/dS ratio as 0.6 (0.425) and 0.364 (0.196) for R and L, respectively. A statistical test using unpaired Wilcoxon rank sum test between dN/dS ratio of R and L suggested significant differences (*P* = 0.0024) among them (Figure 5).

### Mitochondrial DNA Variants Associated With Obesity

Significant associations (*P* > 0.05) with BMI classifications were found for 14 mtDNA variants (Table 3); however, three of these associations were no longer significant after adjusting for age, sex, and maternal haplogroups using multivariate logistic regression (Table 3). In addition, nine mtDNA variants were found when the model was corrected for age, sex, and maternal haplogroups. Thus, a total of 20 SNPs were correlated with obesity, among which 11 were positively (OR > 1) correlated with obesity. The missense variant MT:5460G > A (Ala331Thr) in the *MT-ND2* gene showed the most significant correlation (*P* = 0.009) and was associated with a threefold increased risk of obesity. Among the nine negatively (OR > 1) correlated SNPs, the upstream variant MT:16362T > C in the *MT-TP* gene (encoding microsomal triglyceride transfer protein) showed the most significant (*P* = 0.007; OR = 0.38) negative association with obesity.

**TABLE 3:** Mitochondrial variants associated with obesity in the Kuwaiti population.

**mtDNA variants**	**Gene**	**Consequence**	**Obese F**	**Non-obese F**	**OR (95% CI)**	***P*-value**	OR (95% CI)*	*P*-value*
MT:709G > A	*RNR1*	Non-coding	0.185	0.075	2.8 (1.304–6.012)	0.008	2.4 (1.048–5.495)	0.038
MT:11812A > G	*ND4*	Synonymous	0.065	0.007	9.366 (1.183–74.17)	0.011	10.53 (1.261–87.88)	0.029
MT:14233A > G	*ND6*	Synonymous	0.065	0.007	9.296 (1.174–73.61)	0.012	10.15 (1.212–84.9)	0.032
MT:16362T > C	***TP***	**Upstream**	**0.118**	**0.227**	**0.456 (0.241–0.864)**	**0.017**	**0.377 (0.185–0.766)**	**0.007**
MT:13188C > T	*ND5*	Synonymous	0.053	0.127	0.384 (0.16–0.922)	0.035	0.287 (0.107–0.769)	0.013
MT:16294C > T	*TP*	Upstream	0.144	0.066	2.388 (1.059–5.385)	0.036	2.08 (0.861–5.024)	0.103
MT:13392T > C	*ND5*	Synonymous	0.052	0.007	7.5 (0.925–60.76)	0.038	8.894 (1.014–78.02)	0.048
MT:58T > C	*RNR1*	Upstream	0.02	0.076	0.25 (0.067–0.929)	0.042	0.238 (0.058–0.978)	0.046
MT:64C > T	*RNR1*	Upstream	0.066	0.143	0.421 (0.188–0.943)	0.047	0.327 (0.132–0.809)	0.015
MT:3594C > T	*ND1*	Synonymous	0.086	0.029	3.085 (0.98–9.703)	0.048	3.519 (1.042–11.88)	0.042
MT:13650C > T	*ND5*	Synonymous	0.086	0.029	3.084 (0.98–9.701)	0.048	3.517 (1.043–11.87)	0.042
MT:1018G > A	*RNR1*	Non-coding	0.087	0.03	3.083 (0.979–9.699)	0.048	3.561 (1.054–12.03)	0.04
MT:8292G > A	*TK*	Upstream	0.013	0.06	0.209 (0.043–1.005)	0.049	0.205 (0.038–1.097)	0.064
MT:13500T > C	*ND5*	Synonymous	0.013	0.059	0.21 (0.043–1.007)	0.049	0.228 (0.041–1.269)	0.091
MT:16193C > T	TP	Upstream	0.073	0.022	3.456 (0.943–12.66)	0.056	4.527 (1.105–18.55)	0.035
MT:11719A > G	ND4	Synonymous	0.24	0.343	0.603 (0.358–1.015)	0.064	0.516 (0.273–0.975)	0.041
MT:825T > A	*RNR1*	Non-coding	0.046	0.007	6.372 (0.773–52.48)	0.071	8.891 (1.008–78.42)	0.049
MT:5460G > A	***ND2***	**Missense**	**0.111**	**0.052**	**2.285 (0.916–5.693)**	**0.087**	**3.762 (1.375–10.29)**	**0.009**
MT:2442T > C	*RNR2*	Non-coding	0.06	0.123	0.454 (0.193–1.067)	0.091	0.338 (0.13–0.88)	0.026
MT:769G > A	*RNR1*	Non-coding	0.092	0.037	2.636 (0.923–7.527)	0.093	3.214 (1.045–9.885)	0.041
MT:3847T > C	*ND1*	Synonymous	0.066	0.123	0.508 (0.222–1.165)	0.147	0.359 (0.142–0.91)	0.03
MT:3537A > G	*ND1*	Synonymous	0.006	0.03	0.213 (0.023–1.933)	0.188	0.088 (0.008–0.925)	0.042
MT:7853G > A	*CO2*	Missense	0.006	0.022	0.289 (0.029–2.814)	0.343	0.091 (0.008–0.945)	0.044

*Values after adjustment for age, gender and maternal haplogroup. Abbreviations: F, Frequency; N, number of individuals; OR, odds ratio; CI, confidence intervals. The rows in bold font indicate the most significant (adjusted P-values < 0.01) associations with obesity.

Functional analysis of the consequences of these 20 variants revealed that 12 were located in coding exonic regions, four were in non-coding regions, and four were in gene upstream regions. The SIFT and PolyPhen-2 tools that assess the impact of variants on the protein structure and function predicted these variants as “tolerated” and “benign,” respectively. None of these variants was annotated as pathogenic for obesity by ClinVar, Mitomaster, and Mitomap databases. Nevertheless, the MT:5460G > A missense variant, which is positively correlated with obesity, has been associated with Alzheimer’s disease and Parkinson’s disease (Lin et al., 1992; Schnopp et al., 1996), and the MT:16362T > C, which was negatively correlated with obesity, was shown to be associated with lower mtRNA expression levels and affect uncoupled mitochondrial respiration (Zhou et al., 2017).

Nine SNPs were detected in only one of the BMI groups. Among the four SNPs that were detected in the obese group only, MT:2758G > A (a non-coding variant of *MT-RNR2*) was observed in eight individuals, MT:8468C > T (a synonymous variant of *MT-ATP8*) was observed in six individuals, MT:16320C > T (an upstream variant from *MT-TP*) was observed in six individuals, and MT:93A > G (an upstream variant from *MT-TF*) was observed in six individuals. Five SNPs were detected in the non-obese group only, including MT:10499A > G (a synonymous variant of *MT-ND4L*) observed in six individuals, MT:10609T > C (a missense variant of *MT-ND4L*) observed in four individuals, MT:3197T > C (a non-coding variant of *MT-RNR2*) observed in five individuals, and MT:16288T > C and MT:16359T > C (upstream variants of *MT-TP*) observed in four individuals.

## Discussion

Previous studies have identified mitochondrial haplogroups associated with obesity. Mitochondrial group T was associated with an increased risk of obesity in Austrian (Ebner et al., 2015) and southern Italian (Nardelli et al., 2013) populations. Mitochondrial haplogroups X and H were reported to decrease risk of obesity in Caucasians of northern European origin in the United States (Yang et al., 2011) and Arabs from Kuwait (Eaaswarkhanth et al., 2019), respectively. It should be noted that these significant mitochondrial haplogroup studies did not follow the same approach and that there were differences in the age of the participants (adults versus children), BMI grouping, number of mtDNA variants, and regions studied. Differences in the region studied may explain why some studies, such as those conducted in European–American and African–American populations, found no association between mitochondrial variants and obesity (Grant et al., 2012).

In the present study cohort, the distribution of maternal linage frequency was 75% Western Eurasian, 12.5% African, and 12.5% Asian. This distribution is consistent with previous published distributions in Kuwait (Scheible et al., 2011) as well as neighboring countries, such as Iraq (Al-Zahery et al., 2003) and Saudi Arabia (Abu-Amero et al., 2008).

The frequency of mitochondrial haplogroup R in non-obese group was significantly higher than that in obese group. In the present study, most individuals (85%) in haplogroup R belonged to the R0a clade (Figure 4A and Supplementary Table 2), which is defined by the mutations MT:64C > T, MT:2442T > C, MT:3847T > C, MT:13188C > T, MT:16126T > C, and MT:16362T > C (Abu-Amero et al., 2007). Univariate and multivariate analyses showed that these defining mutations were also negatively correlated with obesity. The same was also observed for MT:11719A > G, which is the defining mutation for an ancestor haplogroup R0. It is important to note that R0a is the most frequent sub-haplogroup in the Arabian Peninsula, with frequency of 5–30%, and it has been speculated that several founders of R0a are present in southern Arabia (Cerny et al., 2011; Scheible et al., 2011). The overall frequency of the R0a haplogroup in our samples was 10%, which is in agreement with the frequency range in the Arabian Peninsula.

The frequency of mitochondrial haplogroup L in the obese group was significantly higher than that in the non-obese group after adjusting for age and sex. Half of the individuals in haplogroup L belonged to the L3 clade (Figure 4B and Supplementary Table 2), which is associated with out-of-Africa migration into Asia (Cabrera et al., 2018). Within the human mtDNA tree, haplogroup L3 encompasses not only many sub-Saharan Africans but also all ancient non-African lineages. The similarity of the age of L3 to its two non-African daughter haplogroups, M and N, suggested that the same process was likely responsible for both the L3 expansion in Eastern Africa and the dispersal of a small group of modern humans out of Africa to settle the rest of the world (Soares et al., 2012). The defining mutations for African subclade L3, MT:769G > A and MT:1018G > A (van Oven and Kayser, 2009), were positively correlated with risk of obesity after adjusting for age, sex, and maternal haplogroup. Mutations from other subclades of haplogroup L were also positively correlated with risk of obesity, including MT:709G > A, MT:8468C > T, MT:3594C > T, MT:13650C > T, MT:825T > A, MT:5460G > A, MT:16320C > T, and MT:93A > G (van Oven and Kayser, 2009).

We observed that mitochondrial haplogroup T, which is known to increase risk of obesity (Nardelli et al., 2013; Ebner et al., 2015), showed a higher frequency in the obese group compared with the non-obese group, but this was not significant. However, its defining mutations, MT:11812A > G and MT:14233A > G, correlated positively with risk of obesity (*P* = 0.029 and *P* = 0.032, respectively). Examination of the mitomap database (Lott et al., 2013) revealed another mutation, MT:10609T > C, which is a marker for a subclade of haplogroup F, which was negatively correlated with risk of obesity. Interestingly, this SNP was associated with athlete status and sprint performance in a Korean population (Hwang et al., 2019).

The metric of evolutionary rate ratio *dN/dS* (ratio of non-synonymous to synonymous substitution rates) indicates how quickly a protein’s constituent amino acids change relative to synonymous changes. A value of <1 indicates purifying selection, =1 indicates evolving neutrally, and >1 indicates positive (diversifying) selection (Spielman and Wilke, 2015). For both the R and L haplogroups that we observed in our study as associated with obesity, the median *dN/dS* ratio was <1 (0.600 and 0.364, respectively), indicating that both the haplogroups undergo purifying selection in the Kuwaiti population; however, the lower ratio in L haplogroup suggested that the L haplogroup (risk effect on obesity) experienced more purifying selection (or negative selection) than the R haplogroup (protective effect on obesity) by purging deleterious mutations in the process of evolution.

We found that several variants located in nicotinamide adenine dinucleotide (NADH) dehydrogenase subunit (*MT-ND1*, *MT-ND4*, and *MT-ND5*) genes, respiratory complex I, and mitochondrial 12S and 16S ribosomal RNA (*MT-RNR1* and *MT-RNR2*) genes were significantly positively or negatively correlated with risk of obesity. In the *MT-RNR2* gene, MT:2758G > A was only identified in the obese group, whereas MT:3197T > C was only identified in the non-obese group. NADH dehydrogenase is required for energy generation in the cell; therefore, variants within its seven encoding genes could result in metabolic disorders including obesity (Flaquer et al., 2014). The *MT-RNR1* gene encodes MOTS-C protein that regulates insulin sensitivity and metabolic homeostasis and plays a protective role against diet-induced obesity (Lee et al., 2015). Furthermore, *MT-RNR2* encodes Humanin, which plays a protective role against oxidative stress (Voigt and Jelinek, 2016). Thus, variants within these genes could potentially interfere with their function, resulting in an increased or decreased risk of obesity.

To prioritize the significant variants identified in our study, we focused on missense mutations leading to amino acid substitutions that were unique to either the obese or non-obese group. The missense variant MT:5460G > A from the *MT-ND2* gene was only positively correlated with obesity. This finding was in agreement with findings from other studies that reported that variants within the *MT-ND2* gene were associated with body fat mass (Yang et al., 2011) and increased BMI (Flaquer et al., 2014). The *MT-ND4L* gene has been associated with obesity (Flaquer et al., 2014) and is a mitochondrial encoding subunit of respiratory complex I. In the present study, the missense mutation MT:10609T > C in the *MT-ND4L* gene was negatively correlated with risk of obesity. Cytochrome *c* oxidase subunit gene 2 (*MT-CO2*), which is an important regulator of the OXPHOS system, was also negatively correlated with risk of obesity. We found that *MT-CO2* harbored a missense mutation, MT:7853G > A, which exhibited a protective role for obesity. A previous study reported that *MT-CO2* (Kraja et al., 2019) and variants within this gene were associated with obesity, but not after adjusting for multiple testing (Liu et al., 2012).

Off-target whole-exome sequencing for the entire mitochondrial genome revealed a good variable coverage depending on the exome capture kit used. The non-uniformity of mitochondrial coverage between the two exome kits could have been due to differences in design and target sequences. This may explain why we observed a higher overlap in variants between sequencing using Sanger technology and sequencing by Nextera Rapid Capture Exome kit compared with sequencing with the TruSeq Exome Enrichment kit (obsolete). Nevertheless, mtDNA variants from both the exome capture kits detected almost all the mtDNA variants identified using indirect whole-genome sequencing of replicated samples. Thus, whole-exome sequencing is a cost- and time-effective alternative for mitochondrial monogenic (Griffin et al., 2014) and association studies (Li et al., 2014) compared with whole-genome sequencing. The reasons for the difference in detection of variants between Sanger and exome variants include the following: (1) low read coverage of exome data at the start and end of the mitochondrial genome (especially when the average mtDNA coverage is <10); (2) repeated poly-C sequencing error using exome data; and (3) the Sanger variant identification pipeline (Eaaswarkhanth et al., 2019) that uses a predicted mtDNA sequence from a sequence browser with manual adjustment could result in a number of false-positive variants.

PCA with the mitochondrial haplogroup profiling from the 288 whole-exome study samples showed a good clustering of haplogroups, which validates the bioinformatics pipeline used in the present study. Moreover, we observed a high concordance (71%) of mitochondrial haplogroup profiling between variants from whole-exome data and the D-loop region data from conventional Sanger sequencing. One sample that was sequenced with whole-genome and exome kits displayed the same assignment of major haplogroup; however, the D-loop Sanger sequencing of the same sample predicted a different haplogroup. This could have an impact on the significant haplogroups identified in previous studies for complex disorders including obesity due to lower resolution or low number of variants used in the studies.

High-throughput NGS of the mitochondrial genome has advantages compared with Sanger sequencing. However, a technical comparison of both the technologies is required to fully understand and unify their results. The present study compared both technologies and found that some variants were only detected by Sanger sequencing and not NGS; however, this discrepancy could be due to low coverage, the whole-exome capture kit design, target sequences, and machine-specific and human error. Nevertheless, other studies observed the same phenomena despite good mitochondrial sequence coverage. For example, variants at positions MT:16183 (Griffin et al., 2014) and MT:523–524 (Park et al., 2017) were only detected by Sanger sequencing. This could have been due to INDEL alignment errors, as its corresponding position in NGS is MT:513. We also found variants that were incorrectly reported between Sanger sequencing and NGS on the same samples at positions MT:302, MT:309, and MT:310 (Park et al., 2017), which could be also due to alignment errors resulting from the complexity of the region. Interestingly, we also observed a sequencing error variant at position MT:3492 that was only detected by whole-genome sequencing and not exome sequencing. This discrepancy may have been an NGS whole-genome sequencing error (Li et al., 2010).

The present study has some limitations. First, the study did not explore mtDNA heteroplasmic variants within the obese and non-obese groups, as these require high coverage sequences. Second, in order to increase the number of study samples, the study utilized data (generated in our previous studies) obtained using two different exome capture kits from Illumina; the Nextera Rapid Capture Exome kit gave a coverage of 50×, while the TruSeq Exome Enrichment kit gave a coverage of mere 8×; having the second one with a very low coverage can weaken the results by not capturing the variants. Third, we divided our study population into obese and nonobese non-obese groups, and the resultant subcohorts were small in size. Despite these limitations, this study paves the way for a larger study to investigate common complex disorders including obesity using whole mtDNA extracted from whole-exome data with greater coverage exome capture kit.

## Conclusion

Indirect whole-exome sequencing of 288 Kuwaiti individuals revealed negative and positive associations of mitochondrial haplogroups R and L, respectively, with obesity. We also identified significantly distributed mtDNA variants among the obese and non-obese groups that were mostly haplogroup-defining mutations. We identified several variants of the NADH dehydrogenase subunit that were significantly positively or negatively correlated with risk of obesity. The present study is the first to utilize whole-exome data to extract entire mitochondrial haplogroups and determine their association with obesity in the Arabian Peninsula.

## Data Availability Statement

The original contributions presented in the study are included in the article/Supplementary Material, further inquiries can be directed to the corresponding author/s.

## Ethics Statement

The studies involving human participants were reviewed and approved by Dasman Diabetes Institute. The patients/participants provided their written informed consent to participate in this study.

## Author Contributions

MD, FA-M, and TT designed and performed the study and wrote the manuscript. RN and MM performed the sequencing. HA and PS performed the statistical analyses. ME, SJ, and PH participated in data analysis. All authors contributed to the article and approved the submitted version.

## Conflict of Interest

The authors declare that the research was conducted in the absence of any commercial or financial relationships that could be construed as a potential conflict of interest.

## Footnotes

1 http://picard.sourceforge.net

2 http://mitomap.org

3 https://www.R-project.org/

## Supplementary Material

Supplementary Figure 1 The complete phylogeny of the R haplogroup in the Kuwaiti population.

Supplementary Figure 2 The complete phylogeny of the L haplogroup in the Kuwaiti population.

Supplementary Table 1 Haplogroup assignments using exome sequence data, Sanger sequencing data and whole genome sequence data.

Supplementary Table 2 Mitochondrial variants used to assign haplogroups in the study cohort of 288 Kuwaiti individuals.

## References

[B1] Abu-Amero K. K. Gonzalez A. M. Larruga J. M. Bosley T. M. Cabrera V. M. (2007). Eurasian and african mitochondrial DNA influences in the saudi arabian population. *BMC Evol. Biol.* 7:32. 10.1186/1471-2148-7-32 17331239 PMC1810519

[B2] Abu-Amero K. K. Larruga J. M. Cabrera V. M. Gonzalez A. M. (2008). Mitochondrial DNA structure in the Arabian Peninsula. *BMC Evol. Biol.* 8:45. 10.1186/1471-2148-8-45 18269758 PMC2268671

[B3] Alsmadi O. John S. E. Thareja G. Hebbar P. Antony D. Behbehani K. (2014). Genome at juncture of early human migration: a systematic analysis of two whole genomes and thirteen exomes from kuwaiti population subgroup of inferred saudi arabian tribe ancestry. *PLoS One* 9:e99069. 10.1371/journal.pone.0103691 PMC4045902 24896259

[B4] Alsmadi O. Thareja G. Alkayal F. Rajagopalan R. John S. E. Hebbar P. (2013). Genetic substructure of kuwaiti population reveals migration history. *PLoS One* 8:e74913. 10.1371/journal.pone.0074913 24066156 PMC3774671

[B5] Al-Zahery N. Semino O. Benuzzi G. Magri C. Passarino G. Torroni A. (2003). Y-Chromosome and MtDNA polymorphisms in Iraq, a crossroad of the early human dispersal and of post-neolithic migrations. *Mol. Phylogenet. Evol.* 28 458–472. 10.1016/s1055-7903(03)00039-3 12927131

[B6] Anderson S. Bankier A. T. Barrell B. G. Debruijn M. H. L. Coulson A. R. (1981). Sequence and organization of the human mitochondrial genome. *Nature* 290 457–465. 7219534 10.1038/290457a0

[B7] Andrews R. M. Kubacka I. Chinnery P. F. Lightowlers R. N. Turnbull D. M. Howell N. (1999). Reanalysis and revision of the cambridge reference sequence for human mitochondrial DNA. *Nat. Genet.* 23 147–147. 10.1038/13779 10508508

[B8] Bouchard C. Perusse L. (1993). “Genetic-Aspects of obesity,” in *Prevention and Treatment of Childhood Obesity*, eds Williams C. L. Kimm S. Y. S., (New York, NY: Annals of the New York Academy of Sciences), 26–35. 10.1111/j.1749-6632.1993.tb18834.x 8267318

[B9] Cabrera V. M. Marrero P. Abu-Amero K. K. Larruga J. M. (2018). Carriers of mitochondrial DNA macrohaplogroup L3 basal lineages migrated back to africa from Asia around 70,000 years ago. *BMC Evol. Biol.* 18:98. 10.1186/s12862-018-1211-4 29921229 PMC6009813

[B10] Calvo S. E. Compton A. G. Hershman S. G. Lim S. C. Lieber D. S. Tucker E. J. (2012). Molecular diagnosis of infantile mitochondrial disease with targeted next-generation sequencing. *Sci. Transl. Med.* 4:118ra10. 10.1126/scitranslmed.3003310 PMC3523805 22277967

[B11] Cerny V. Mulligan C. J. Fernandes V. Silva N. M. Alshamali F. Non A. (2011). Internal diversification of mitochondrial haplogroup r0a reveals post-last glacial maximum demographic expansions in South Arabia. *Mol. Biol. Evol.* 28 71–78. 10.1093/molbev/msq178 20643865

[B12] Chang C. C. Chow C. C. Lcam T. Vattikuti S. Purcell S. M. Lee J. J. (2015). Second-Generation plink: rising to the challenge of larger and richer datasets. *Gigascience* 4:7. 10.1186/s13742-015-0047-8 PMC4342193 25722852

[B13] Chaubey G. Metspalu M. Kivisild T. Villems R. (2007). Peopling of South Asia: investigating the caste-tribe continuum in india. *Bioessays* 29 91–100. 10.1002/bies.20525 17187379

[B14] Chinnery P. F. Elliott H. R. Hudson G. Samuels D. C. Relton C. L. (2012). Epigenetics, epidemiology and mitochondrial DNA diseases. *Int. J. Epidemiol.* 41 177–187. 10.1093/ije/dyr232 22287136 PMC3304530

[B15] Clayton D. A. (2000). Transcription and replication of mitochondrial DNA. *Hum. Reprod.* 15 (Suppl. 2), 11–17. 10.1093/humrep/15.suppl_2.11 11041509

[B16] Delmiro A. Rivera H. Garcia-Silva M. T. Garcia-Consuegra I. Martin-Hernandez E. Quijada-Fraile P. (2013). Whole-Exome sequencing identifies a variant of the mitochondrial Mt-Nd1 gene associated with epileptic encephalopathy: west syndrome evolving to lennox-gastaut syndrome. *Hum. Mutat.* 34 1623–1627. 10.1002/humu.22445 24105702

[B17] Diroma M. A. Calabrese C. Simone D. Santorsola M. Calabrese F. M. Gasparre G. (2014). Extraction and annotation of human mitochondrial genomes from 1000 genomes whole exome sequencing data. *BMC Genom.* 15:S2. 10.1186/1471-2164-15-S3-S2 25077682 PMC4083402

[B18] Eaaswarkhanth M. Melhem M. Sharma P. Nizam R. Al Madhoun A. Chaubey G. (2019). Mitochondrial DNA D-Loop sequencing reveals obesity variants in an arab population. *Appl. Clin. Genet.* 12 63–70. 10.2147/tacg.s198593 31213875 PMC6541754

[B19] Ebner S. Mangge H. Langhof H. Halle M. Siegrist M. Aigner E. (2015). Mitochondrial haplogroup t is associated with obesity in austrian juveniles and adults. *PLos One* 10:e0135622. 10.1371/journal.pone.0135622 26322975 PMC4556186

[B20] Fernandes V. Alshamali F. Alves M. Costa M. D. Pereira J. B. Silva N. M. (2012). The arabian cradle: mitochondrial relicts of the first steps along the Southern route out of Africa. *Am. J. Hum. Genet.* 90 347–355. 10.1016/j.ajhg.2011.12.010 22284828 PMC3276663

[B21] Fernandez-Sanchez A. Madrigal-Santillan E. Bautista M. Esquivel-Soto J. Morales-Gonzalez A. Esquivel-Chirino C. (2011). Inflammation, oxidative stress, and obesity. *Int. J. Mol. Sci.* 12 3117–3132. 21686173 10.3390/ijms12053117 PMC3116179

[B22] Flaquer A. Baumbach C. Kriebel J. Meitinger T. Peters A. Waldenberger M. (2014). Mitochondrial genetic variants identified to be associated with BMI in adults. *PLos One* 9:e105116. 10.1371/journal.pone.0105116 25153900 PMC4143221

[B23] Grant S. F. A. Glessner J. T. Bradfield J. P. Zhao J. Tirone J. E. Berkowitz R. I. (2012). Lack of relationship between mitochondrial heteroplasmy or variation and childhood obesity. *Int. J. Obesity* 36 80–83. 10.1038/ijo.2011.206 22005716

[B24] Greenberg B. D. Newbold J. E. Sugino A. (1983). Intraspecific nucleotide-sequence variability surrounding the origin of replication in human mitochondrial-DNA. *Gene* 21 33–49. 10.1016/0378-1119(83)90145-2 6301949

[B25] Griffin H. R. Pyle A. Blakely E. L. Alston C. L. Duff J. Hudson G. (2014). Accurate mitochondrial DNA sequencing using off-target reads provides a single test to identify pathogenic point mutations. *Genet. Med.* 16 962–971. 10.1038/gim.2014.66 24901348 PMC4272251

[B26] Hosgood H. D. Liu C. S. Rothman N. Weinstein S. J. Bonner M. R. Shen M. (2010). Mitochondrial DNA copy number and lung cancer risk in a prospective cohort study. *Carcinogenesis* 31 847–849. 10.1093/carcin/bgq045 20176654 PMC2864414

[B27] Hwang I. W. Kim K. Choi E. J. Jin H. J. (2019). Association of mitochondrial haplogroup F with physical performance in korean population. *Genom. Informat.* 17:e11. 10.5808/gi.2019.17.1.e11 30929412 PMC6459174

[B28] John S. E. Antony D. Eaaswarkhanth M. Hebbar P. Channanath A. M. Thomas D. (2018). Assessment of coding region variants in kuwaiti population: implications for medical genetics and population genomics. *Sci. Rep.* 8:16583. 10.1038/s41598-018-34815-8 PMC6224454 30409984

[B29] Kivisild T. (2015). Maternal ancestry and population history from whole mitochondrial genomes. *Investigat. Genet.* 6:3. 10.1186/s13323-015-0022-2 25798216 PMC4367903

[B30] Knoll N. Jarick I. Volckmar A. L. Klingenspor M. Illig T. Grallert H. (2014). Mitochondrial DNA variants in obesity. *PLos One* 9:e94882. 10.1371/journal.pone.0094882 24788344 PMC4008486

[B31] Kraja A. T. Liu C. Y. Fetterman J. L. Graff M. Have C. T. Gu C. (2019). Associations of mitochondrial and nuclear mitochondrial variants and genes with seven metabolic traits. *Am. J. Hum. Genet.* 104 112–138. 30595373 10.1016/j.ajhg.2018.12.001 PMC6323610

[B32] Lee C. Zeng J. Drew B. G. Sallam T. Martin-Montalvo A. Wan J. X. (2015). The mitochondrial-derived peptide Mots-c promotes metabolic homeostasis and reduces obesity and insulin resistance. *Cell Metab.* 21 443–454. 10.1016/j.cmet.2015.02.009 25738459 PMC4350682

[B33] Leigh J. W. Bryant D. (2015). Popart: full-feature software for haplotype network construction. *Methods Ecol. Evol.* 6 1110–1116. 10.1111/2041-210x.12410

[B34] Li H. (2013). Aligning sequence reads, clone sequences and assembly contigs with BWA-MEM. *ArXiv* [preprint] 3:13033997.

[B35] Li H. Handsaker B. Wysoker A. Fennell T. Ruan J. Homer N. (2009). The sequence alignment/map format and samtools. *Bioinformatics* 25 2078–2079. 10.1093/bioinformatics/btp352 19505943 PMC2723002

[B36] Li M. K. Schonberg A. Schaeferd M. Schroeder R. Nasidze I. Stoneking M. (2010). Detecting heteroplasmy from high-throughput sequencing of complete human mitochondrial DNA genomes. *Am. J. Hum. Genet.* 87 237–249. 10.1016/j.ajhg.2010.07.014 20696290 PMC2917713

[B37] Li S. T. Besenbacher S. Li Y. R. Kristiansen K. Grarup N. Albrechtsen A. (2014). Variation and association to diabetes in 2000 full mtdna sequences mined from an exome study in a danish population. *Eur. J. Hum. Genet.* 22 1040–1045. 10.1038/ejhg.2013.282 24448545 PMC4350597

[B38] Lin F. H. Lin R. Wisniewski H. M. Hwang Y. W. Grundkeiqbal I. Healylouie G. (1992). Detection of point mutations in codon-331 of mitochondrial nadh dehydrogenase subunit-2 in alzheimer brains. *Biochem. Biophys. Res. Commun.* 182 238–246. 10.1016/s0006-291x(05)80136-6 1370613

[B39] Liu C. Y. Yang Q. Hwang S. J. Sun F. Z. Johnson A. D. Shirihai O. S. (2012). Association of genetic variation in the mitochondrial genome with blood pressure and metabolic traits. *Hypertension* 60:949. 10.1161/hypertensionaha.112.196519 22949535 PMC3753106

[B40] Loogvali E. L. Roostalu U. Malyarchuk B. A. Derenko M. V. Kivisild T. Metspalu E. (2004). Disuniting uniformity: a pied cladistic canvas of mtdna haplogroup h in eurasia. *Mol. Biol. Evol.* 21 2012–2021. 10.1093/molbev/msh209 15254257

[B41] Lott M. T. Leipzig J. N. Derbeneva O. Xie H. M. Chalkia D. Sarmady M. (2013). mtDNA variation and analysis using mitomap and mitomaster. *Curr. Protocols Bioinform.* 1 1–6. 10.1002/0471250953.bi0123s44 PMC4257604 25489354

[B42] McKenna A. Hanna M. Banks E. Sivachenko A. Cibulskis K. Kernytsky A. (2010). The genome analysis toolkit: a mapreduce framework for analyzing next-generation dna sequencing data. *Genome Res.* 20 1297–1303. 10.1101/gr.107524.110 20644199 PMC2928508

[B43] McLaren W. Gil L. Hunt S. E. Riat H. S. Ritchie G. R. S. (2016). The ensembl variant effect predictor. *Genome Biol.* 17:122. 10.1186/s13059-016-0974-4 PMC4893825 27268795

[B44] Nardelli C. Labruna G. Liguori R. Mazzaccara C. Ferrigno M. Capobianco V. (2013). Haplogroup T is an obesity risk factor: mitochondrial dna haplotyping in a morbid obese population from Southern Italy. *Biomed Res. Int.* 2013:631082. 10.1155/2013/631082 PMC3713591 23936828

[B45] Naukkarinen J. Heinonen S. Hakkarainen A. Lundbom J. Vuolteenaho K. Saarinen L. (2014). Characterising metabolically healthy obesity in weight-discordant monozygotic twins. *Diabetologia* 57 167–176. 10.1007/s00125-013-3066-y 24100782

[B46] Ng M. Fleming T. Robinson M. Thomson B. Graetz N. Margono C. (2014). Global, regional, and national prevalence of overweight and obesity in children and adults during 1980-2013: a systematic analysis for the global burden of disease study 2013. *Lancet* 384 766–781. 24880830 10.1016/S0140-6736(14)60460-8 PMC4624264

[B47] Park S. Cho S. Seo H. J. Lee J. H. Kim M. Y. Lee S. D. (2017). Entire mitochondrial DNA sequencing on massively parallel sequencing for the Korean population. *J. Korean Med. Sci.* 32 587–592. 10.3346/jkms.2017.32.4.587 28244283 PMC5334155

[B48] Patowary A. Nesbitt R. Archer M. Bernier R. Brkanac Z. (2017). Next generation sequencing mitochondrial dna analysis in autism spectrum disorder. *Autism Res.* 10 1338–1343. 10.1002/aur.1792 28419775 PMC5573912

[B49] Picardi E. Pesole G. (2012). Mitochondrial genomes gleaned from human whole-exome sequencing. *Nat. Methods* 9 523–524. 10.1038/nmeth.2029 22669646

[B50] Ramachandrappa S. Farooqi I. S. (2011). Genetic approaches to understanding human obesity. *J. Clin. Invest.* 121 2080–2086. 10.1172/jci46044 21633175 PMC3104766

[B51] Samuels D. C. Han L. Li J. Sheng Q. H. Clark T. A. Shyr Y. (2013). Finding the lost treasures in exome sequencing data. *Trends Genet.* 29 593–599. 10.1016/j.tig.2013.07.006 23972387 PMC3926691

[B52] Scheible M. Alenizi M. Sturk-Andreaggi K. Coble M. D. Ismael S. Irwin J. A. (2011). Mitochondrial DNA control region variation in a kuwaiti population sample. *Forensic Sci. Int. Genet.* 5 E112–E113. 21555259 10.1016/j.fsigen.2011.04.001

[B53] Schnopp N. M. Kosel S. Egensperger R. Graeber M. B. (1996). Regional heterogeneity of Mtdna heteroplasmy in parkinsonian brain. *Clin. Neuropathol.* 15 348–352. 8937782

[B54] Soares P. Achilli A. Semino O. Davies W. Macaulays V. Bandelt H. J. (2010). The archaeogenetics of Europe. *Curr. Biol.* 20 R174–R183. 20178764 10.1016/j.cub.2009.11.054

[B55] Soares P. Alshamali F. Pereira J. B. Fernandes V. Silva N. M. Afonso C. (2012). The expansion of Mtdna haplogroup L3 within and out of Africa. *Mol. Biol. Evol.* 29 915–927. 10.1093/molbev/msr245 22096215

[B56] Sorensen T. I. A. Holst C. Stunkard A. J. (1998). Adoption study of environmental modifications of the genetic influences on obesity. *Int. J. Obesity* 22 73–81. 10.1038/sj.ijo.0800548 9481603

[B57] Spielman S. J. Wilke C. O. (2015). The relationship between Dn/Ds and scaled selection coefficients. *Mol. Biol. Evol.* 32 1097–1108. 25576365 10.1093/molbev/msv003 PMC4379412

[B58] Stunkard A. J. Harris J. R. Pedersen N. L. McClearn G. E. (1990). The body-mass index of twins who have been reared apart. *New Engl. J. Med.* 322 1483–1487. 2336075 10.1056/NEJM199005243222102

[B59] Tang S. Wang J. Zhang V. W. Li F. Y. Landsverk M. Cui H. (2013). Transition to next generation analysis of the whole mitochondrial genome: a summary of molecular defects. *Hum. Mutat.* 34 882–893. 23463613 10.1002/humu.22307

[B60] Thareja G. John S. E. Hebbar P. Behbehani K. Thanaraj T. A. Alsmadi O. (2015). Sequence and analysis of a whole genome from kuwaiti population subgroup of persian ancestry. *BMC Genom.* 16:92. 10.1186/s12864-015-1233-x 25765185 PMC4336699

[B61] van Oven M. Kayser M. (2009). Updated comprehensive phylogenetic tree of global human mitochondrial DNA variation. *Hum. Mutat.* 30 E386–E394. 18853457 10.1002/humu.20921

[B62] Veronese N. Stubbs B. Koyanagi A. Vaona A. Demurtas J. Schofield P. (2018). Mitochondrial genetic haplogroups and incident obesity: a longitudinal cohort study. *Eur. J. Clin. Nutrit.* 72 587–592. 29386643 10.1038/s41430-018-0097-y

[B63] Voigt A. Jelinek H. F. (2016). Humanin: a mitochondrial signaling peptide as a biomarker for impaired fasting glucose-related oxidative stress. *Physiol. Rep.* 4:e12796. 10.14814/phy2.12796 PMC4873641 27173674

[B64] Wagner M. Berutti R. Lorenz-Depiereux B. Graf E. Eckstein G. Mayr J. A. (2019). Mitochondrial DNA mutation analysis from exome sequencing-a more holistic approach in diagnostics of suspected mitochondrial disease. *J. Inherit. Metab. Dis.* 42 909–917. 31059585 10.1002/jimd.12109

[B65] Weissensteiner H. Pacher D. Kloss-Brandstatter A. Forer L. Specht G. Bandelt H. J. (2016). Haplogrep 2: mitochondrial haplogroup classification in the era of high-throughput sequencing. *Nucleic Acids Res.* 44 W58–W63. 27084951 10.1093/nar/gkw233 PMC4987869

[B66] Wong L. J. C. (2013). Next generation molecular diagnosis of mitochondrial disorders. *Mitochondrion* 13 379–387. 23473862 10.1016/j.mito.2013.02.001

[B67] World Health Organization [WHO], (2018). *Noncommunicable Diseases Country Profiles.* Geneva: World Health Organization.

[B68] Wortmann S. B. Zweers-Van Essen H. Rodenburg R. J. T. Van Den Heuvel L. P. De Vries M. C. (2009). Mitochondrial energy production correlates with the age-related BMI. *Pediatric Res.* 65 103–108. 10.1203/PDR.0b013e31818d1c8a 19096353

[B69] Yang T. L. Guo Y. Shen H. Lei S. F. Liu Y. J. Li J. (2011). Genetic association study of common mitochondrial variants on body fat mass. *PLoS One* 6:e21595. 10.1371/journal.pone.0021595 21747914 PMC3126834

[B70] Zhou H. Nie K. Qiu R. Xion J. Shao X. Wang B. (2017). Generation and bioenergetic profiles of cybrids with East Asian mtDNA haplogroups. *Oxid Med. Cell Longev.* 2017:1062314. 10.1155/2017/1062314 29093766 PMC5637837

