# Supplementary material for: Delineation of Mitochondrial DNA Variants From Exome Sequencing Data and Association of Haplogroups With Obesity in Kuwait
Source: Front Genet. 2021 Feb 11;12:626260. doi: 10.3389/fgene.2021.626260 (PMC7920096; doi:10.3389/fgene.2021.626260)
Supplement: Supplementary Figure 1 — The complete phylogeny of the R haplogroup in the Kuwaiti population. [file Data_Sheet_1.PDF]

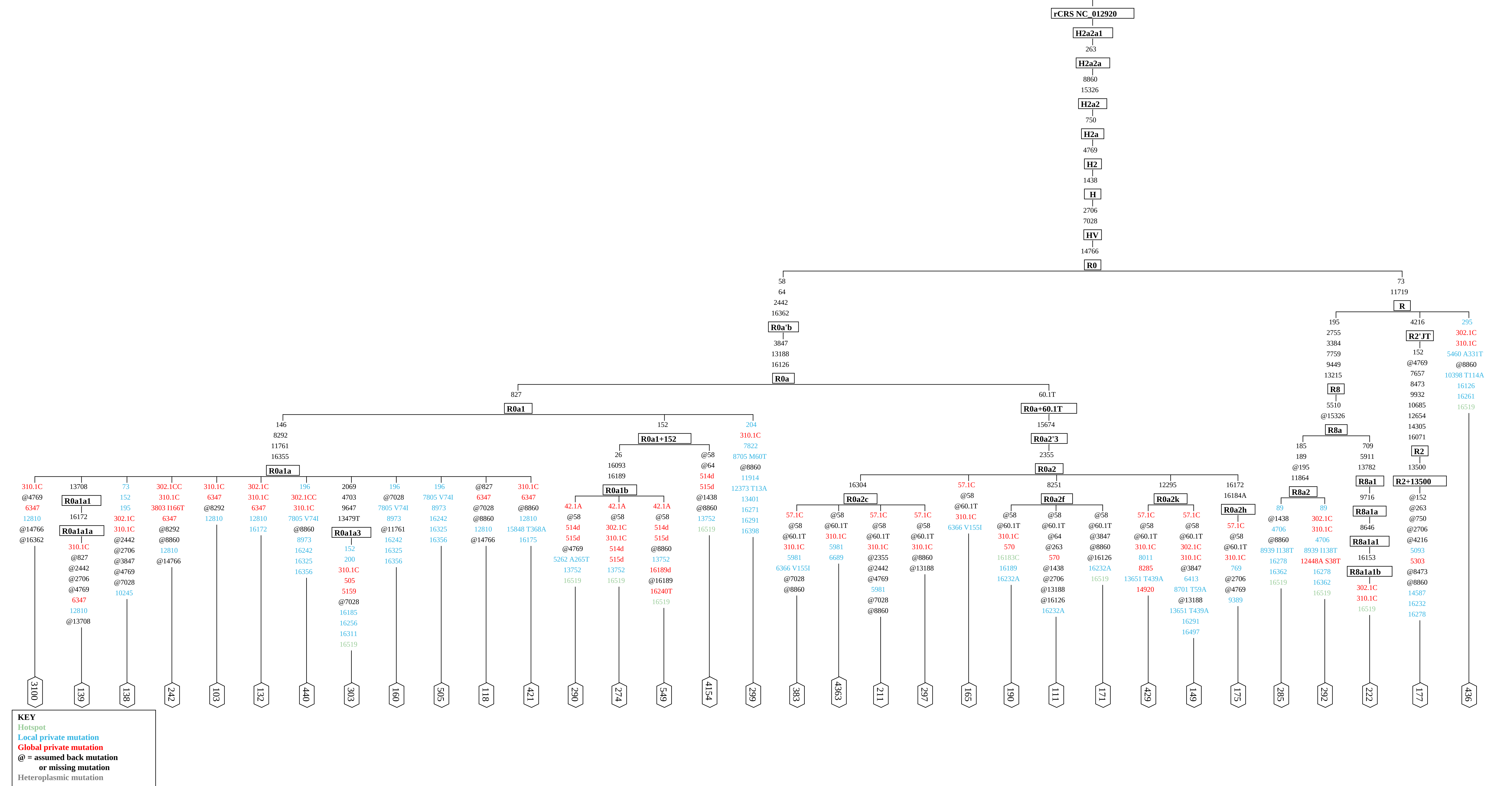

rCRS NC_012920
H2a2a1
263
H2a2a
8860
15326
H2a2
750
H2a
4769
H2
1438
H
2706
7028
HV
14766
R0
58
64
2442
16362
R0'b
3847
13188
16126
R0a
827
R0a1
146
8292
11761
16355
R0a1a
152
R0a1+152
26
16093
16189
R0a1b
60.1T
R0a+60.1T
15674
R0a2'3
2355
R0a2
16304
R0a2c
8251
R0a2f
12295
R0a2k
16172
16184A
R0a2h
73
11719
R
195
2755
3384
7759
9449
13215
R8
5510
@15326
R8a
185
189
@195
11864
R8a2
709
5911
13782
R8a1
9716
R8a1a
8646
R8a1a1
16153
R8a1a1b
4216
R2'JT
152
@4769
7657
8473
9932
10685
12654
14305
16071
R2
13500
R2+13500
13708
R0a1a1
16172
R0a1a1a
2069
4703
9647
13479T
R0a1a3
3100
139
138
242
103
132
440
303
160
505
118
421
290
274
549
4154
299
383
4363
211
297
165
190
111
171
429
149
175
285
292
222
177
436
KEY
Hotspot
Local private mutation
Global private mutation
@ = assumed back mutation
or missing mutation
Heteroplasmic mutation
