# Supplementary figures and images for: Delineation of Mitochondrial DNA Variants From Exome Sequencing Data and Association of Haplogroups With Obesity in Kuwait
Source: Front Genet. 2021 Feb 11;12:626260. doi: 10.3389/fgene.2021.626260 (PMC7920096; doi:10.3389/fgene.2021.626260)

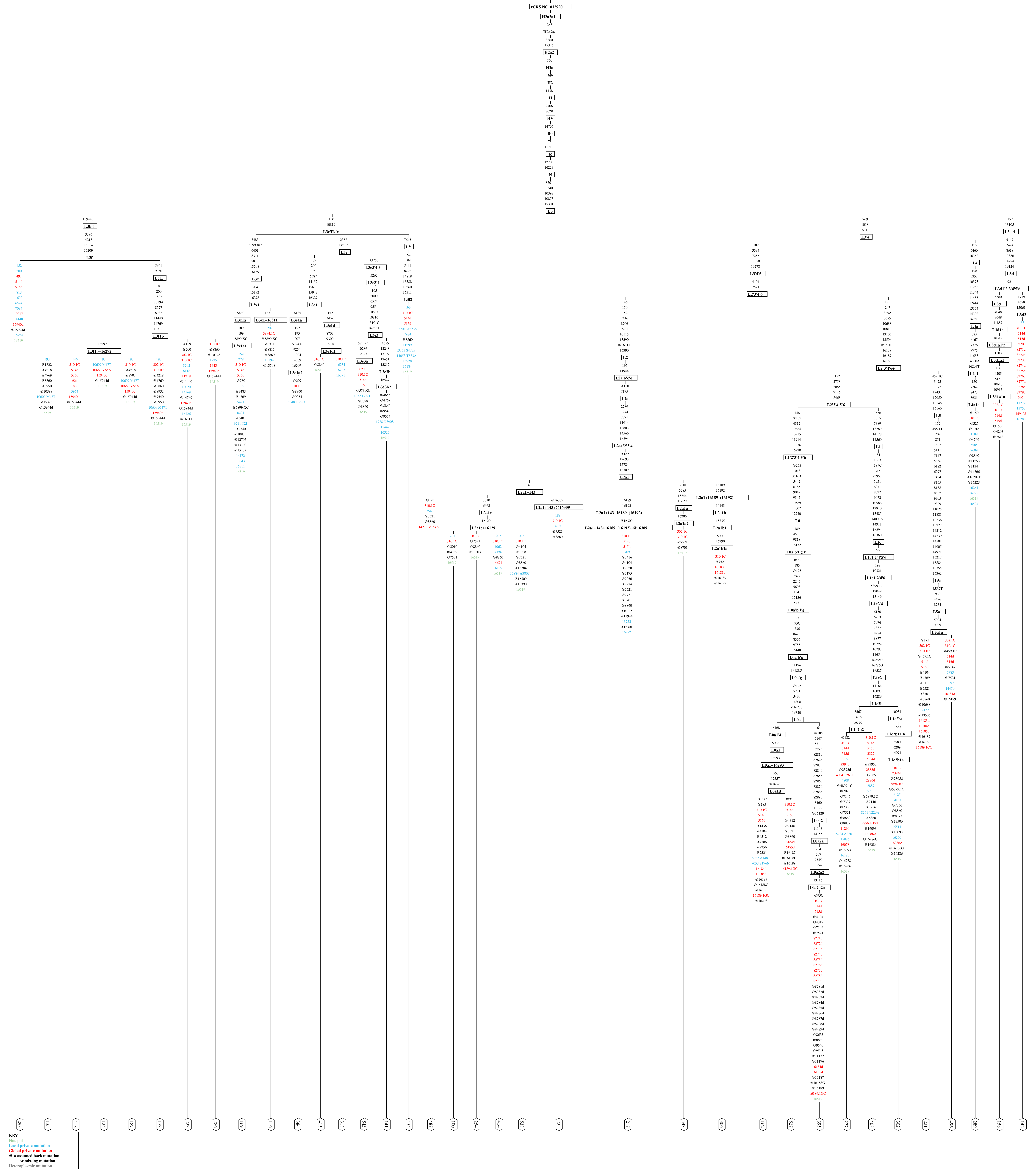

Supplement: Supplementary Figure 2 — The complete phylogeny of the L haplogroup in the Kuwaiti population. [file Data_Sheet_2.PDF]
